# Mitochondrial Iron Metabolism: The Crucial Actors in Diseases

**DOI:** 10.3390/molecules28010029

**Published:** 2022-12-21

**Authors:** Geyan Duan, Jianjun Li, Yehui Duan, Changbing Zheng, Qiuping Guo, Fengna Li, Jie Zheng, Jiayi Yu, Peiwen Zhang, Mengliao Wan, Cimin Long

**Affiliations:** 1 CAS Key Laboratory of Agro-Ecological Processes in Subtropical Region, Hunan Provincial Key Laboratory of Animal Nutritional Physiology and Metabolic Process, National Engineering Laboratory for Pollution Control and Waste Utilization in Livestock and Poultry Production, Institute of Subtropical Agriculture, Chinese Academy of Sciences, Changsha 410125, China; 2 College of Advanced Agricultural Sciences, University of Chinese Academy of Sciences, Beijing 100049, China; 3 College of Animal Science and Technology, Hunan Agricultural University, Changsha 410128, China

**Keywords:** iron homeostasis, mitochondrial dysfunction, diseases

## Abstract

Iron is a trace element necessary for cell growth, development, and cellular homeostasis, but insufficient or excessive level of iron is toxic. Intracellularly, sufficient amounts of iron are required for mitochondria (the center of iron utilization) to maintain their normal physiologic function. Iron deficiency impairs mitochondrial metabolism and respiratory activity, while mitochondrial iron overload promotes ROS production during mitochondrial electron transport, thus promoting potential disease development. This review provides an overview of iron homeostasis, mitochondrial iron metabolism, and how mitochondrial iron imbalances-induced mitochondrial dysfunction contribute to diseases.

## 1. Introduction

Iron exerts an essential role in living organisms. On one hand, iron is a component of heme (e.g., myoglobin, hemoglobin, myeloperoxidase, cytochrome proteins, nitric oxide synthetases), iron-sulfur clusters (e.g., mitochondrial aconitase, coenzyme Q10, respiratory complexes I–III), or other functional groups (e.g., hypoxia inducible factor prolyl hydroxylases) incorporated into proteins as cofactors. These iron-containing proteins contribute to various biological processes, such as oxygen transport and energy metabolism [1]. On the other hand, iron is involved in oxidation-reduction reactions by readily shuttling between the oxidized ferric (Fe^3+^) and the reduced ferrous (Fe^2+^) forms. The reactions are required for a number of fundamental biologic processes. Notably, the cellular redox equilibrium can be easily disrupted by catalytic amounts of iron, thus resulting in the generation of toxic reactive oxygen species (ROS) and oxidative stress [2,3]. Under oxidative stress, mitochondria (the cellular energy centers) are impaired, leading to impaired energy state and potential disease development [4,5]. As such, iron has become a key target of interest in the progression and treatment of diseases related to dysfunction in mitochondria and energy metabolism. Preventing the dysfunctional role of iron in energy metabolism may help prevent or delay related metabolic diseases [6]. Therefore, this review emphasizes the importance of iron homeostasis in mitochondrial function and energy metabolism and discusses the diseases that are related to imbalances in iron homeostasis, mitochondrial dysfunction, and impaired energy metabolism.

## 2. Cellular Iron Absorption, Utilization, and Homeostasis

**Figure 1:**
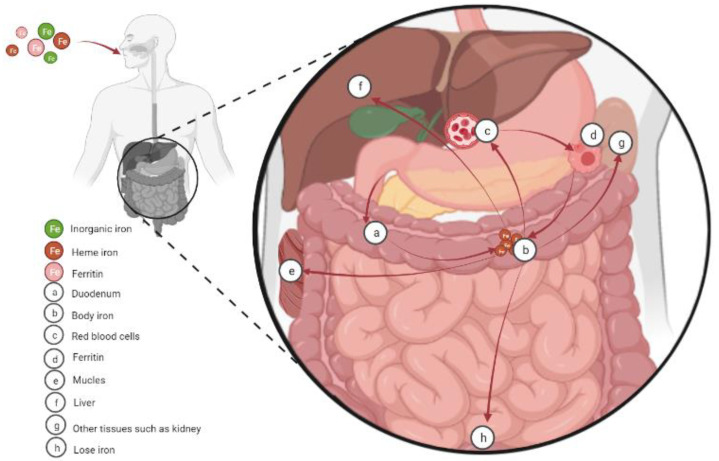
Cellular iron absorption, utilization, and homeostasis. Dietary iron is absorbed in the duodenum in the following three forms: inorganic (mainly present in the oxidized form Fe^3+^), heme, and ferritin. Then the iron is transported to the body. Body iron is mainly present in the hemoglobin of red blood cells and developing erythroid cells (at least 2.1 g in humans). In addition, macrophages (up to 600 mg) and the myoglobin of muscles (~300 mg) also contain significant amounts of iron, and the liver stores the excess body iron (~1 g). Other tissues also contain lower, but not negligible, quantities of iron. Finally, mammals lose iron from sloughing of mucosal and skin cells or during bleeding.

The molecular mechanism of cellular iron absorption and metabolism has been well characterized and shown in lots of reviews (Figure 1) [7,8]. Therefore, we only discuss it briefly in this review before discussing the role of iron in energy metabolism. The hemoglobin (at least 2.1 g in humans) of red blood cells and developing erythroid cells is the main place where body iron exists. In addition, body iron also exists in macrophages (up to 600 mg), the myoglobin of muscles (~300 mg), and the liver (~1 g). Notably, lower, but not negligible, quantities of iron also exist in other tissues. On the other hand, the main ways for iron excretion from the body are sloughing of mucosal and skin cells or during bleeding, but the underlying regulated mechanism remains unclear. In the presence of physiological pH and oxygen, dietary iron mainly exists in the form of highly insoluble iron Fe (III), while the iron transport system absorbs ferrous Fe (II) ions, which are very unstable and rapidly oxidized to trivalent iron [9,10]. On this basis, balance is maintained by the tight control of dietary absorption in the duodenum [11,12]. Dietary iron is absorbed in the following three forms: inorganic (mainly present in the oxidized form Fe^3+^), heme, and ferritin. Prior to intestinal uptake, dietary inorganic iron (Fe^3+^ form) must be reduced to the Fe^2+^ form by the cytochrome b on the duodenal enterocyte membrane [13,14]. Then, with the help of divalent metal transporter 1 (DMT1) on the membrane, the Fe^2+^ is further transported into intestinal epithelial cells [15]. Iron (Fe^2+^) taken up by enterocytes has the following four fates: (1) stored in ferritin in its Fe^3+^ form; (2) used directly as a cofactor by cytosolic proteins; (3) transported into mitochondria; and (4) transported out of the cell [7]. Unlike dietary inorganic iron, the mechanisms for uptake of dietary heme and ferritin are less well understood. However, after it is liberated, iron obtained from dietary heme and ferritin enters a common pathway similar to inorganic iron in the enterocyte [16].

With the help of ferroportin1 (FPN1), a known iron transmembrane efflux protein in vertebrate cells, intracellular iron is exported out of the cell [17,18,19]. Another important way to remove intracellular iron is by extracellular vesicles (specifically, by exosomes), hence protecting cells from ferroptotic cell death [20]. To guard dissociative iron against oxidative damage to cells, excess cellular iron is stored in ferritin [21]. Exported iron is scavenged by transferrin, which maintains Fe^3+^ in a redox-insert state and delivers it into tissues by the ubiquitously expressed transferrin receptor 1 (TFR1) [22]. Under normal conditions, iron exists in the bloodstream mainly in the form of transferrin-bound iron, which is not redox active and does not produce extrahepatic iron overload. Once plasma iron exceeds the carrying capacity of transferrin, iron and transferrin are not tightly bound to form non-transferrin-bound iron (NTBI), which is taken up by tissues (such as the heart, pancreas, and liver) through endocytosis [23], thus giving rise to tissue damage [24].

The level of body iron needs close regulation since imbalances between the two oxidation states of iron produce ROS [25]. The maintenance of iron homeostasis is largely modulated by the iron regulatory protein (IRP)-iron response element (IRE) system, which is a relatively simple and ubiquitous post-transcriptional regulatory loop. In response to alterations in the levels of intracellular iron, this system can regulate the expression of post-translational ferritin and transferrin receptors and alter the synthesis of pivotal iron metabolic proteins [26,27]. That is, when cellular iron levels are low, IRP rescues cellular iron deficiency by the following two mechanisms: (1) binding of IRP to the 5′UTR of mRNA blocks mRNA translation of key proteins associated with iron storage and export; (2) binding of IRP to the 3′UTR of mRNA elevates mRNA translation of key proteins related to iron uptake. The opposite effect occurs when cellular iron levels are high [28]. Thus, when iron supply exceeds cell demand, the IRE-IRP switch minimizes further iron uptake via TfR1 and facilitates the storage of excess iron in newly synthesized ferritin to reach cellular iron homeostasis.

## 3. Iron and Energy Metabolism

Mitochondrial function is traditionally associated with energy supply for all cell compartments [29]. However, fresh insights into the relationship between mitochondrial energy metabolism and mitochondrial iron levels necessitates an expansion of the concept. The mitochondrion requires sufficient amounts of iron to maintain its normal physiologic function, since iron is the most prevalent metal inside the mitochondrial matrix and serves to facilitate the complex redox chemistry of the electron transport chain [7,30]. Once imported into mitochondria, iron is stored in the mitochondrial ferritin, or used for the biosynthesis of heme [31] and the biogenesis of iron-sulfur cluster (ISC or Fe-S) [32,33]. Both of them facilitate oxidation-reduction reactions and are essential components of enzymes involved in electron transport [34,35]. Specifically, mitochondrial iron-containing proteins that are implicated in the electron-transport chain include heme-containing proteins (succinate dehydrogenase, cytochrome c, cytochrome c oxidase, and cytochrome bc1), the ISC-containing proteins (nicotinamide adenine dinucleotide (NADH) ubiquinone oxidoreductase, Rieske iron-sulfur protein, subunits of succinate dehydrogenase, biotin synthase, lipoic acid synthase, and aconitase), and iron-ion cofactor-containing proteins (iron monooxy-genases and dioxygenases) [36]. Notably, the unique redox properties of iron allow for efficient electron transfer, accompanied by the generation of ROS. Accordingly, insufficient or excessive levels of mitochondrial iron can impair the synthesis of Fe-S cluster and heme, induce mitochondrial dysfunction, and cause oxidative stress, consequently affecting mitochondrial ATP production via the tricarboxylic acid (TCA) and/or glycolysis [37,38,39].

## 4. Mitochondrial Iron and Diseases

All mammalian cells possess mitochondria, and mitochondrial function is required for normal cell physiological processes. Consequently, these cells are vulnerable to diseases related to failure of mitochondrial iron homeostasis and consequent mitochondrial dysfunction [37], as shown in Table 1. These diseases are discussed in detail in the following sections.

### 4.1. Cardiovascular Disease

Heart failure is a pressing public health problem with no curative treatment currently available. According to the report, heart failure is caused by the changes in mitochondrial iron homeostasis and mitochondrial function [50,51,52]. Mitochondrial iron is involved in the energy metabolism of the heart and is a fundamental element of cardiomyocyte viability and contractility [40,53]. On the one hand, systemic iron deficiency decreases mitochondrial function, leading to iron deficiency in cardiomyocytes even without anemia [54,55,56]. Recent work in mice [57] and patients with heart failure [40] has been clarified that mitochondrial function is reduced when intracellular iron is deficient, which leads to severe heart failure and is associated with cardiomyocyte injury [41]. The myocardium cannot provide sufficient blood flow. On the other hand, increased heme iron intake and body iron stores have been reported to be strongly associated with cardiovascular risk [58,59,60]. Excess iron can lead to impaired vascular function, aggravating atherosclerosis, arrhythmia, and heart failure [61]. ROS production is also catalyzed by excess iron, which causes lipid peroxidation and organelle damage [62]. This leads to cardiomyocyte death and fibrosis, ultimately leading to impaired systolic and diastolic function. Support for this theory derives from observations of increased mitochondrial iron levels in patients with heart failure [42]. The best documented example has been clearly shown in a human genetic disease, namely, cardiomyopathy of Friedreich’s ataxia (FRDA) [42]. Recent work in patients [63] and in mouse models [64,65] provides evidence that this disease is characterized by significant accumulation of iron inside the mitochondria, extensive mitochondrial dysfunction, and oxidative damage [42,66]. Luckily reducing mitochondrial iron is able to protect the heart by inhibiting oxidative stress [67]. Interestingly, the cardiac phenotype observed in FRDA is partially ameliorated in response to combined therapy with the mitochondria-permeable iron chelator deferiprone and an antioxidant [68,69], supporting the role of mitochondrial iron in the pathophysiology of cardiac dysfunction. Further confirmation for a relationship between mitochondrial iron accumulation and heart failure comes from the findings that deletion of mitochondrial ATP binding cassette transporter B8 in the heart inhibits iron export from this organelle and results in mitochondrial iron overload and subsequently increased oxidative stress [70].

**Figure 2:**
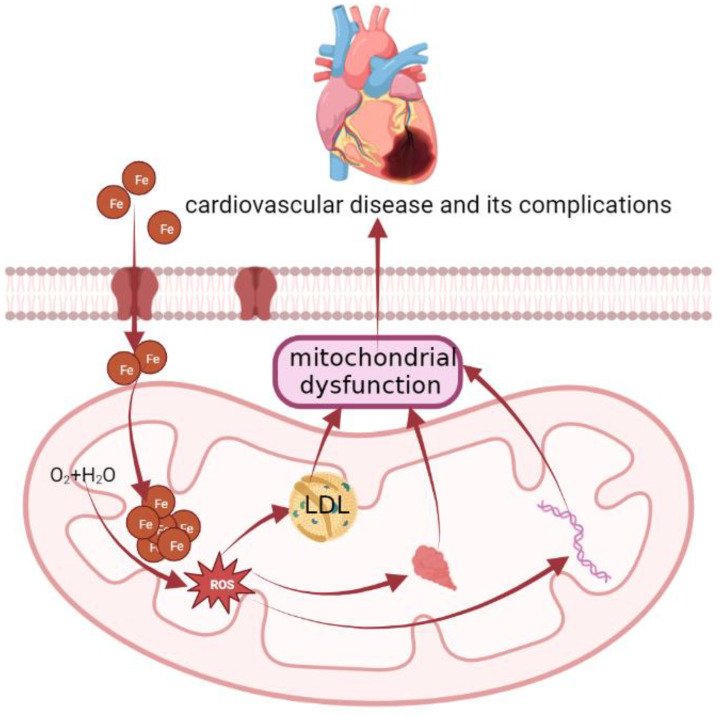
The molecular mechanism of cardiovascular disease. Concerning the mechanisms, the elevation in mitochondrial iron levels that results in heart failure is likely mediated by potential disruption of Fe-S cluster biogenesis and by an ROS-dependent mechanism. Iron is specially involved in the formation of atherosclerosis by catalyzing the generation of ROS, promoting the peroxidation of the lipid and protein parts of lipoproteins, and forming oxidized low-density lipoprotein (LDL). ROS causes mitochondrial damage by attacking mitochondrial DNA and mitochondrial proteins, impairing mitochondrial aerobic metabolism, and mitochondrial dysfunction will also increase the production of ROS, thereby forming a vicious circle, which ultimately manifests as cardiovascular disease and its complications. Meanwhile, oxidized-LDL can induce macrophages to form foam cells and promote the development of atherosclerosis.

Concerning the mechanisms (Figure 2), the elevation in mitochondrial iron levels that results in heart failure is likely mediated by potential disruption of Fe-S cluster biogenesis and by an ROS-dependent mechanism [71]. Iron is specially involved in the formation of atherosclerosis by catalyzing the generation of free radicals, promoting the peroxidation of the lipid and protein parts of lipoproteins, and forming oxidized low-density lipoprotein (LDL). ROS causes mitochondrial damage by attacking mitochondrial DNA and mitochondrial proteins and impairing mitochondrial aerobic metabolism, and mitochondrial dysfunction will also increase the production of ROS, thereby forming a vicious circle, which ultimately manifests as cardiovascular disease and its complications [72,73]. Meanwhile, oxidized-LDL can induce macrophages to form foam cells and promote the development of atherosclerosis [74,75,76]. Then, to make matters worse, mitochondrial antioxidant enzymes are significantly reduced in patients with heart failure compared with normal subjects [77].

Similar to iron overload, iron deficiency can also be detrimental to the heart, an organ with high energy demands. There is validation that iron deficiency is present in approximately 30%~50% of patients with chronic heart failure [78]. Heart failure symptoms in the patient population can be improved by intravenous iron supplementation, which has been a recommended treatment for patients with heart failure with iron deficiency [79,80].

### 4.2. Liver Disease

The liver, the main site for iron storage, is the main target organ of iron overload-induced injury. When the iron storage and antioxidant capacity of the liver is exceeded, iron overload can lead to liver fibrosis, cirrhosis, and even hepatoma, as seen in β-thalassemia and hereditary hemochromatosis [43,81]. In addition, other chronic liver diseases such as viral hepatitis, nonalcoholic fatty liver disease, and alcoholic liver disease, are also related to liver iron overload [43]. Liver iron overload-induced oxidative stress may be a contributing mechanism for the progression of these diseases [43,82]. The liver is susceptible to oxidative damage by its intermediate metabolites during the process of metabolic detoxification. Excessive pro-oxidative forms of iron in the parenchymal cells of the liver promote oxidative damage, triggering lipid peroxidation [83]. Iron-driven injury of hepatocytes can lead to paracrine induction of hepatic stellate cells and portal myofibroblasts through lipid peroxidation byproducts, leading to increased collagen deposition, fibrosis and long-term micronodular cirrhosis and hepatocellular carcinoma [84]. In explanation of the reason for iron accumulation in the liver, recent studies have suggested that pathogenic factors related to the underlying liver disease may contribute the iron overload by directly affecting the expression of hepcidin (for autocrine downregulation of FPN expression to reduce iron export) [85].

### 4.3. Muscle Atrophy

Muscle atrophy, also called sarcopenia, is characterized by loss of skeletal muscle mass [86] and can be induced by aging [87] and various chronic diseases [88,89]. Recent evidence points to a strong relationship between mitochondrial iron accumulation and muscle atrophy [90], possibly manifested as a decrease in type II muscle fiber content [91,92]. Previous work on aged rats shows that, due to alterations in iron metabolism, increased iron accumulation and decreased muscle mass occur in parallel [44,93,94,95]. The adaptive downregulation in IRP2 results in a decreased expression of TFR1 (an iron transporter) and an increased expression of ferritin (an iron storage protein), which constitutes a proposed mechanism that may explain the accelerated iron accumulation in skeletal muscle of aged rats [44]. In line with these findings, recent studies showed that ablation of TFR1 in satellite cells impedes skeletal muscle regeneration through activation of ferroptosis [96]. Further support for this mechanism drives from observations of higher ferritin levels in women with sarcopenia or sarcopenic obese people [97,98,99]. Another potential mechanism for the iron accumulation is related to the lower expression of FPN and the upregulation of genes related to iron uptake (such as DMT1 and Zip14) [100]. In addition, an animal model of disuse atrophy was used to further our understanding of the underlying mechanisms for the iron accumulation. The researchers found that iron accumulation induced by acute muscle atrophy was related to extensive oxidative stress after reloading in skeletal muscles of aged rats [100]. Oxidative stress induced by excessive iron causes muscle damage [101,102]. In support, sarcopenia and oxidative stress in skeletal muscles of mice were induced in response to iron administration [103]. Despite these interesting findings, our understanding of the precise molecular mechanism of iron-induced muscle atrophy is incomplete. Upon further investigation, the E3 ubiquitin ligase mediated by the reduction of Akt-forkhead box O3a signaling by oxidative stress is a contributing mechanism for the iron-induced skeletal muscle atrophy [86]. It is manifested in the promotion of protein degradation and inhibition of protein synthesis [86,103,104]. In 2019, it was revealed for the first time that the iron metabolism regulatory molecule Hemojuvelin (HJV or HFE2) is a protective gene that inhibits the occurrence of Duchenne muscular dystrophy and senile muscle atrophy. The molecular regulation mechanism of HJV dependent on TGFb/SMAD2/3 pathway was elucidated, and the important physiological role of HJV in protecting muscle and resisting muscle fiber aging was further explored [105]. This achievement provides a new target for the prevention and treatment of muscle atrophy diseases.

### 4.4. Obesity and Diabetes

Obesity and diabetes are becoming one of the most pressing health issues facing society. Several studies provide strong evidence for the correlation between dysregulated iron homeostasis and obesity as well as diabetes [29,106,107]. The liver and adipose tissues of obese participants had higher iron concentrations [108,109,110,111]. Iron accumulation and the related oxidative stress contribute to the pathophysiology of obesity and its related metabolic disturbances, such as type 2 diabetes mellitus [108]. Iron accumulation increases ROS through Fenton reaction, leading to mitochondrial dysfunction in adipocytes. This toxic effect on β cells leads to defects in insulin synthesis and secretion [45,112,113]. Hyperglycemia exacerbates iron accumulation, promotes oxidative stress and the development of type 2 diabetes [114]. In support, adipogenesis and mitochondrial biosynthesis are greatly inhibited when transferrin is knocked down or iron is chelated by using deferoxamine (DFO) [115,116], thereby inhibiting the development of obesity in diabetic states [107]. Consistently, adiposity can be ameliorated in response to DFO (100 mg/kg body weight), accompanied by increased insulin sensitivity in ob/ob mice [107]. On the contrary, lipolysis is promoted when adipocytes are treated with either iron or transferrin [117]. The status and development of obesity and diabetes can be ameliorated when body iron content is reduced to an appropriate level [106]. However, contradictory results are reported by other studies, which indicate that iron deficiency increases the risk of developing diabetes in obese individuals [118]. Therefore, the relationship between body iron content and obesity is still a topic of debate and warrants further investigation.

### 4.5. Kidney Disease

Iron and iron-triggered oxidative stress and mitochondrial dysfunction are thought to be involved in the progression of multiple models of acute kidney injury [119,120,121]. Patients with chronic kidney disease (CKD) experience significant changes in iron balance and tissue distribution due to elevated iron losses, decreased iron absorption, and impaired mobilization of iron from stores [122]. If the iron metabolism is unbalanced, the accumulation of iron in the kidney and the increase of urinary iron concentration or iron deficiency will cause kidney damage and related complications [6,123,124]. Tubular cell lysosomal iron accumulation has been shown in patients with CKD [46], which is most likely due to excessive iron content, which catalyzes the formation of oxygen free radicals, disrupts mitochondrial oxidative metabolism, and leads to renal cell damage. According to the Fenton reaction, abnormal accumulation of iron creates oxidative stress. On the other hand, renal tubular epithelial cells have high energy demands and, therefore, have a large number of mitochondria, making them susceptible to oxidative stress [119]. In rat kidneys, iron in the form of myoglobin has been reported to generate oxidative stress, leading to mitochondrial dysfunction through lipid peroxidation of mitochondrial membranes, which leads to pro-inflammatory cells in a rat model of acute cerebral ischemia Factor production [123].

Ferroptosis, a new form of regulated cell death identified in recent years, is involved in the initiation and progression of diverse kidney diseases, such as renal ischemia-reperfusion injury, renal cell carcinoma, and acute kidney injury [125,126]. Unlike other types of known regulated cell death (e.g., pyroptosis, necrosis, autophagy, and apoptosis), ferroptosis is characterized by the iron-dependent overwhelming accumulation of lipid hydroperoxides and augmented mitochondrial membrane density [127]. The latest research demonstrated that mitochondrial iron overload can accelerate the process of ferroptosis [128]. Concerning the mechanism for iron overload-induced ferroptosis, recent studies using a model of aristolactam I-induced ferroptosis reported that Fe2+ overload-mediated mitochondrial ROS over-release would activate lipid peroxidation and inhibit the antioxidant system by inhibiting nuclear factor erythroid 2-related factor 2-heme oxygenase 1/glutathione peroxidase 4 pathway, which enhanced ferroptosis [129].

### 4.6. Neurodegenerative Disease (NDDs)

The brain is a metabolically active place compared to other organs [130]. Neuronal mitochondrial respiration accounts for about 20% of total oxygen consumption [131]. Cortical neurons in the human brain utilize approximately 4.7 billion ATP molecules produced by mitochondria per second to perform biological functions such as synaptic assembly, generation of action potentials, and synaptic transmission [132]. It has been reported that mitochondrial iron accumulation plays an important role in the initiation and progression of NDDs, such as Alzheimer’s disease (AD) and Parkinson’s disease (PD) [133]. In detail, iron overload promotes mitochondrial dysfunction and catalyzes the production of ROS that triggers oxidative stress in the brain, resulting in neurological damage [134]. It has been reported that mitochondrial dysfunction is a common pathogenic feature of NDDs such as AD and PD [135,136]. Ferritin is a precursor of iron accumulation [137]. The two subunits of ferritin, L- ferritin (FTL) and H- ferritin (FTH), are essential for iron storage in vertebrate cells [138]. Compared with the liver, which mainly contains FTL, the brain and heart have more high iron oxidation activity, so it mainly contains FTH ferritin with significant antioxidant activity [139,140,141]. Differed from physiological ferritin, studies have shown that ferritin structures in NDDs are in the form of magnetite crystals [142]. This rare magnetic structure could help visualize brain tissue for the diagnosis of NDDs [143].

**Figure 3:**
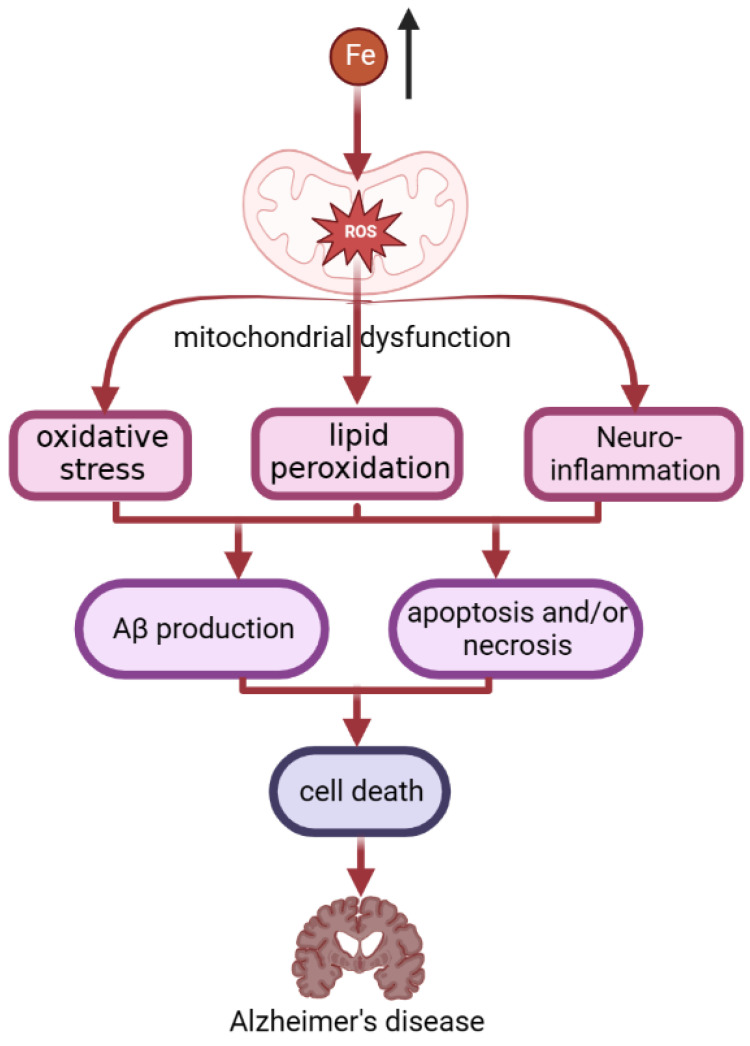
The process of iron accumulation in AD patients. In AD, iron accumulation induces oxidative stress, lipid peroxidation, and inflammatory responses by disrupting mitochondrial function, and inducing ROS production. The combined effects of oxidative stress, lipid peroxidation, and neuroinflammation lead to the production of amyloid-beta (A*β*). A*β* can induce lipid peroxidation in the presence of iron ions. Through these mechanisms, iron accumulation induces apoptosis and/or necrosis, thus leading to cell death. This finally leads to becoming an AD patient.

It is estimated that 10% of the world’s population may currently be affected by AD [144]. Patients with AD have diffuse accumulation of iron in the cerebral cortex and hippocampus, and the content of iron in senile plaques increases slightly [47]. Specifically, in AD (Figure 3), iron accumulation induces oxidative stress, lipid peroxidation, and inflammatory responses by disrupting mitochondrial function, depleting ATP, and inducing ROS production [135]. The combined effects of oxidative stress, lipid peroxidation and neuroinflammation lead to the production of amyloid-beta (A*β*) [132]. Through these mechanisms, iron accumulation induces apoptosis and/or necrosis, thus leading to cell death [135]. In addition, A*β* can induce lipid peroxidation in the presence of iron ions [145], as manifested by the increased expression of lipoxygenase in the brain of AD patients [146]. Knockout of lipoxygenase reduces iron-induced lipid peroxidation, which in turn reduces A*β* deposition in AD mouse brain and improves behavioral performance [147].

Among other NDDs, PD is the second most common in people over 60 [148]. Focal accumulation of iron in the substantia nigra has been reported in patients with PD [48]. Iron is involved in the formation of α-synuclein aggregates in intracellular inclusions, called Lewy bodies, leading to synaptic dysfunction and disruption of axonal transport [149], which is a hallmark of PD. In murine models of PD, α-synuclein expression can be regulated to ameliorate PD injury by increasing mitochondrial ferritin [150,151]. Moreover, decreased mitochondrial complex I activity is observed in mitochondria isolated from human brain tissues and peripheral cells of sporadic PD patients, indicating an impairment of mitochondrial function [152]. Subsequently, such mitochondrial dysfunction may result in IRP1 activation, upregulated expression of DMT1 and TFR1, elevated uptake of iron, and elevated production of ROS [153]. The mitochondrial iron-specific changes in human and rodent models of PD have been demonstrated by a number of studies. For instance, mitochondrial iron uptake and the production of ROS were increased in SH-SY5Y dopaminergic neuroblastoma cells treated with rotenone (a mitochondrial complex I inhibitor) [154,155]. Further evidence comes from observations of the accumulation of transferrin in dopamine neurons (with much of it accumulating in the mitochondria) in a rodent rotenone model of PD [156].

### 4.7. Cancers

**Figure 4:**
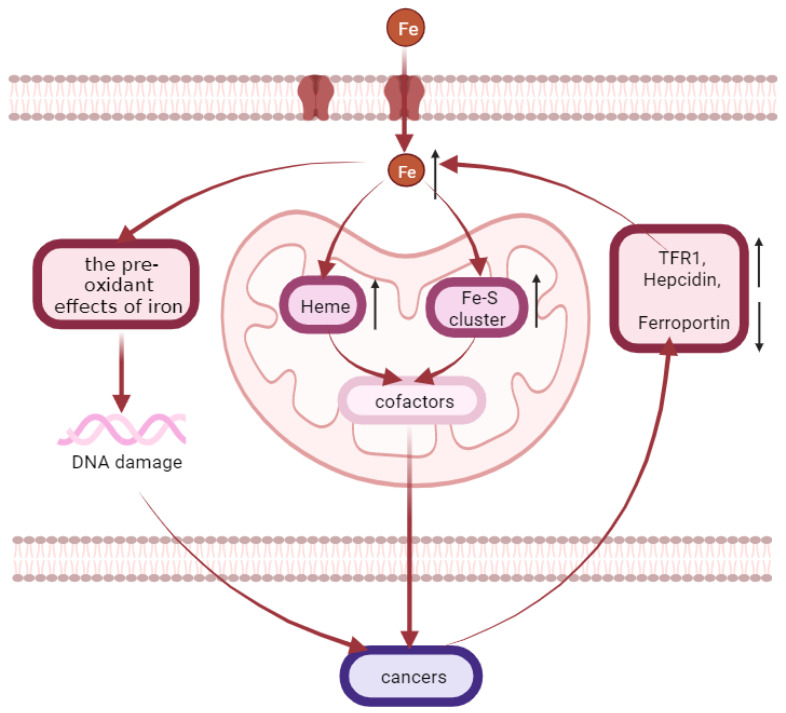
Iron accumulation is related to the occurrence of various cancers. Iron overload is related to the occurrence of various cancers such as liver, colon, rectum, lung, esophagus, and bladder cancers. There are two well-defined mechanisms of cancer development induced by iron excess. One is associated with the pre-oxidant effects of iron, which can lead to DNA damage and subsequently promote oncogenesis. The other one is that more iron may be imported to mitochondria of cancer cells during rapid cell proliferation, in order to produce heme and Fe-S cluster and to satisfy increasing demands for these cofactors. Meanwhile, the expression of iron homeostasis proteins associated with iron accumulation is altered in multiple cancer cell types, such as an elevated expression of the iron uptake-related protein TFR1, a reduced expression of the iron export-related protein ferroportin, and an elevated production of hepcidin.

Iron overload is related to the occurrence of various cancers such as liver, colon, rectum, lung, esophagus and bladder cancers (Figure 4) [157,158] because iron is needed in all stages of tumor development, survival, proliferation and metastasis [159]. There are two well-defined mechanisms of cancer development induced by iron excess [160,161]. One is associated with the pre-oxidant effects of iron, which can lead to DNA damage and subsequently promote oncogenesis [162]. The dependence of cancer cells on iron to maintain their rapid growth rate constitutes the other mechanism [161,163]. During rapid cell proliferation, more iron may be imported to mitochondria of cancer cells, in order to produce heme and ISC and to satisfy increasing demands for these cofactors [164]. For instance, the rates of heme-synthesis are elevated in non-small cell lung cancer cells compared to normal nonmalignant lung cells [165]. Intriguingly, the expression of iron homeostasis proteins associated with iron accumulation is altered in multiple cancer cell types, such as an elevated expression of the iron uptake-related protein TFR1, a reduced expression of the iron export-related protein FPN, and an elevated production of hepcidin [160,161,166,167,168]. Tumor growth and survival can be greatly influenced by altered expression of these proteins. Evidence for this is provided by observations found in breast cancer that a high expression of FPN and low expression of hepcidin predicts a favorable prognosis, while a low expression of FPN is related to metastatic progression and reduced survival [169,170,171]. In addition, the expression of mitoferrin-2 (related to mitochondrial iron uptake) is altered in head and neck cancers [172]. The demand for iron in cancer cells is an important strategy for the anti-cancer targeting of chelating agents. Iron chelators affect the initiation, growth, proliferation, and metastasis of cancer cells by targeting different stages of disease progression, including associated iron metabolic pathways and iron-containing proteins [160,173]. The first iron chelator for clinical trials is desferrioxamine (DFO) [174,175], which was originally used as a treatment for iron overload [176]. It can also target ferritin through autophagy degradation [177]. Quercetin can not only effectively form complexes with iron, but also induce iron deficiency behaviors in cancer cells, such as induction of transferrin receptor-1 and iron regulatory protein-2 expression and decreased ferritin expression. In addition, quercetin can regulate the expression of iron metabolism genes in rats and reduce the expression of DMT1, Dcytb, FPN, and hepcidin. This reduces the level of iron absorption [178]. In addition, the new iron chelator CN128 has great potential in the treatment of clinical skin cancer, with good oral bioavailability and tissue distribution [179]. It is worth mentioning that, according to a new study, the increase in iron can promote estrogen-induced carcinogenesis by producing additional ROS [49]. This may be a new breakthrough in the treatment of cancer.

## 5. Summary

**Figure 5:**
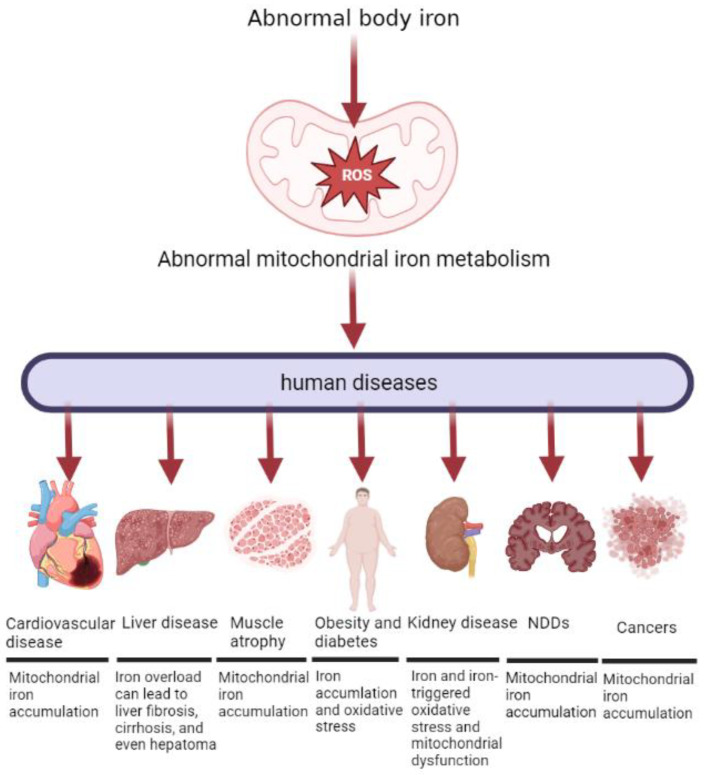
Abnormal mitochondrial iron metabolism can cause different diseases in the human body. Both insufficient and excessive levels of iron can be detrimental to mitochondrial function. Mitochondria are found in human cells, and normal cellular physiology depends on mitochondrial function. Consequently, these cells are vulnerable to diseases associated with failure of mitochondrial iron homeostasis and consequent mitochondrial dysfunction. The diseases described in the paper are: Sideroblastic anemia, Cardiovascular disease, liver disease, muscle atrophy, obesity and diabetes, kidney disease, Neurodegenerative diseases, and cancers.

The literature reviewed here indicates that iron has important physiological and pathological significance in the body. Disorders of mitochondrial iron metabolism underlie the pathogenesis of many diseases (Figure 5). In detail, mitochondrial iron deficiency or overload can result in dysfunctional mitochondrial synthesis of heme and/or ISC, causing mitochondrial dysfunction and consequent oxidative damage. This may lead to further downstream signals to induce various diseases. However, information is limited to the optimal iron treatment strategy for the diseases. In the near future, more efforts should be made to find better diagnostic parameters for accurately gauging iron status and to take measures to maintain the mitochondrial iron balance, ultimately promoting the healthy growth of the body.

## Figures and Tables

**Table 1:** Classification of diseases based on major consequences of changes in iron levels.

Disease Examples or Models	Changes in Iron Level	Major Consequences
Patients [40] and mice [41] with heart failure	Intracellular iron is deficient	Severe heart failure
Cardiomyopathy of Friedreich’s ataxia [42]	Mitochondrial iron levels increased	Cardiomyocyte death and fibrosis, impaired systolic and diastolic function.
β-thalassemia and hereditary hemochromatosis [43]	Iron overload	Liver fibrosis, cirrhosis, and even hepatoma
Aged rats muscle atrophy [44]	Mitochondrial iron accumulation	Muscle mass decreased
Patients with obesity and diabetes [45]	Iron accumulation	Mitochondrial dysfunction in adipocytes causes toxic effects on β cells leading to defects in insulin synthesis and secretion
Patients with chronic kidney disease [46]	Tubular cell lysosomal iron accumulation	Renal cell damage
AD [47]	Diffuse accumulation of iron in the cerebral cortex and hippocampus, and the content of iron in senile plaques increases slightly	Apoptosis and/or necrosis, thus leading to cell death
PD [48]	Focal accumulation of iron in the substantia nigra	The formation of α-synuclein leaded to synaptic dysfunction and disruption of ax-onal transport
Breast cancer [49]	Iron overload^1^	Promoting cancer cell proliferation

^1^ The concentration of transferrin was obtained by immunological method. The transferrin saturation was calculated as the ratio of serum iron to transferrin concentration (TF) multiplied by a factor of 70.9. When the saturation of transferrin exceeds 80%, non-transferrin-bound iron (NTBI) is produced, which is highly reactive and harmful to cells (iron overload).

## Data Availability

Not applicable.

## Sample Availability

Samples of the compounds are not available from the authors.
